# Alterations in HIV-1 gp120 V3 Region Are Necessary but Not Sufficient for Coreceptor Switching in CRF07_BC in China

**DOI:** 10.1371/journal.pone.0093426

**Published:** 2014-03-27

**Authors:** Lei Zhang, Liying Ma, Zheng Wang, Yan Wang, Jing Zhang, Haining Wang, Yiming Shao

**Affiliations:** Division of Research on Virology and Immunology, State Key Laboratory for Infectious Disease Prevention and Control, National Center for AIDS/STD Control and Prevention (NCAIDS), China CDC, Beijing, China; South Texas Veterans Health Care System and University of Texas Health Science Center at San Antonio, United States of America

## Abstract

The most predominant HIV-1 strains in China's current epidemic is the Circulating Recombinant Form 07_BC (CRF07_BC). CRF07_BC is mainly considered as a CCR5-tropic (R5) virus, since CXCR4-tropic (X4) viruses have thus far not been found in this subtype, and the molecular determinants of coreceptor switching remain unknown. To investigate the mechanisms underlying coreceptor requirement in CRF07_BC viruses, we characterized a panel of pNL4-3-based chimeric viruses with mutated V3 loop regions derived from an HIV-1 CRF07_BC infectious clone pXJDC13. Among 17 chimeric viruses, seven were dual-tropic and induced syncytium formation in MT-2 cells. Two amino acid insertions between positions 13 and 14, as well as arginine substitution at position 11 or 16 (IG insertion and P16R mutation or MG insertion and S11R mutation), conferred the chimeric viruses CXCR4-tropic features, which were same as subtype C X4 viruses. Next, to construct CRF07_BC X4 variants, mutated V3 loops were cloned into the CRF07_BC infectious clone pXJDC13. These V3 loops, which in the pNL4-3 backbone conferred chimeric viruses with CXCR4-using ability, abrogated infectivity completely in the CRF07_BC pXJDC13 genetic background. Similarly, IG insertion or MG insertion and S11R mutation dramatically diminished or completely abolished viral infectivity in other envelopes of subtype C or CRF07_BC. These results suggest that the effects of IG insertion and P16R mutation or MG insertion and S11R mutation on CXCR4 usage are context dependent, and additional mutations elsewhere in the envelope are needed to compensate for these fitness-reducing alterations.

## Introduction

The envelope glycoproteins of human immunodeficiency virus type 1 (HIV-1) serve to mediate viral entry into cells. This process requires the sequential interaction of gp120 with the CD4 receptor and coreceptors, such as CCR5 or CXCR4 [1]. Viruses that only use either CCR5 or CXCR4 coreceptors are termed R5- or X4-tropic, respectively, while R5X4 dual-tropic viruses can use both. HIV-1 infection is almost always established with R5 viruses, which predominate during the acute and asymptomatic phases of infection [2]. The appearance of X4 and/or R5X4 viruses is associated with a more rapid decline of CD4+ cells in peripheral blood and faster progression to AIDS [3]–[7]. Although R5 viruses typically persist into late stages of disease, CXCR4- or dual-tropic viruses emerge in approximately 50% of individuals infected with subtype B viruses, and coreceptor switching is also reported in subtype A, C, D, CRF01_AE, and CRF02_AG infections [8]–[12].

HIV-1 coreceptor tropism can play an important role in disease treatment. Small-molecule antagonists that bind CCR5 and inhibit gp120-CCR5 interaction through an allosteric mechanism have been developed, including Maraviroc (MVC), an FDA-approved CCR5 antagonist [13]. MVC is now available as an option for first-line antiretroviral therapy (ART), and is also being considered as prophylaxis against HIV-1 transmission. Patients should ideally be tested for HIV-1 coreceptor tropism prior to initiating such therapy. However, as viral phenotype assays are costly and time-consuming [14], coreceptor usage prediction through sequence analysis may serve as an attractive alternative [15]–[17]. Accurate, timely, and cost-effective means of determining coreceptor tropism will be increasingly important as CCR5 antagonist based drugs become more popularized in developing countries.

A gene region of particular importance to coreceptor usage prediction is the V3 loop of the HIV-1 envelope. V3 typically consists of 35 amino acids and plays a number of important biological roles, including CCR5/CXCR4 tropism [18], [19]. Specific amino acid variations within the V3 loop have been identified to influence coreceptor usage. Amino acid positions 11 and 25 are essential for distinguishing R5 from X4/R5X4 viruses, with the latter often containing positively charged amino acids at these positions [20]–[22]. In addition, in subtype C, X4 viruses are often found to have insertions of two amino acids between positions 13 and 14 and substitutions of arginine at the GPGQ crown [8], [23]–[26]. Currently, almost all genotypic prediction methods rely on V3 sequence features alone, including the 11/25 rule, B-PSSM, C-PSSM, geno2pheno, and CoRSeqV3-C [15]–[17], [27], [28]. However, most existing studies on coreceptor tropism are conducted in subtype B viruses, and while some determinants of CXCR4 usage have been reported in subtype C, little is known for other genotypes.

Our study aims to clarify the genetic signatures of coreceptor switching in the Circulating Recombinant Form CRF07_BC. CRF07_BC is the most predominant HIV-1 strains in China's current epidemic, accounting for approximately 35.5% of all infections [29]. This recombinant strain is the descendent of Thai subtype B′ and Indian subtype C, and was first found in China among injection drug users (IDU) in Xinjiang region in 1997. There are 10 breakpoints within the CRF07_BC genome, and the envelope region is mainly derived from subtype C, except for the first 84 nucleotides derived from subtype B [30], [31]. Clinical isolates in CRF07_BC use coreceptor CCR5 exclusively, and so far CXCR4-tropic viruses have not been found [32], [33]. All existing CRF07_BC sequences in the Los Alamos HIV Sequence Database are predicted to be CCR5-tropic [34]. Yet little research has been conducted specifically on CRF07_BC viruses. In particular, the way in which CRF07_BC viruses bind to and interact with different coreceptors is poorly understood.

Does the coreceptor prediction methods developed on other subtypes also work in CRF07_BC? Do CRF07_BC CXCR4-tropic viruses share the same characteristics in the V3 region with X4 variants in other subtypes? Can CRF07_BC X4 variants be constructed by mutagenesis according to the V3 characteristics of subtype B and C X4 viruses? Here we systematically studied the impact of X4-like V3 features on the CRF07_BC infectious clone pXJDC13. We first characterized the molecular determinants on coreceptor switching within the V3 loop of CRF07_BC, using pNL4-3-based chimeras. The CRF07_BC CXCR4-tropic V3 loops shared the same features with subtype C X4 virus, but not subtype B. As well, the CXCR4-using V3 genotype completely abrogated the infectivity of CRF07_BC V3 mutants. Before coreceptor-specific mutations could occur within V3, additional mutations elsewhere in the envelope gene were needed to maintain viral fitness. These findings serve as a foundation for future research into therapeutics and vaccines that specifically target CRF07_BC infections.

## Materials and Methods

### Cells

293T and TZM-b1 cell lines were maintained in Dulbecco's modified Eagle's medium (DMEM) supplemented with 10% FBS, 50 µg/ml gentamicin and 25 mM HEPES. Ghost.CD4.CCR5 and Ghost.CD4.CXCR4 cells were maintained in DMEM supplemented with 10% FBS, 500 µg/ml G418, 100 µg/ml hygromycin, penicillin-streptomycin, and 1 µg/ml puromycin. MT-2 cells were maintained in RPMI 1640 with 10% FBS.

### V3 sequence analysis for HIV-1 subtype B, C, and CRF07_BC

As CRF07_BC is a recombinant genotype based on subtypes B and C, all available HIV-1 patient-derived V3 sequences for subtype B, C, and CRF07_BC were retrieved from the Los Alamos HIV Sequence Database (http://www.hiv.lanl.gov/content/index). One sequence was retrieved per patient, resulting in 635 subtype B sequences (594 for R5 and 41 for X4), 359 subtype C sequences (339 for R5 and 20 for X4) and 859 CRF07_BC sequences. Sequence variability at each position was determined using the Los Alamos HIV Sequence Database Entropy-One tool (http://www.hiv.lanl.gov/content/sequence/ENTROPY/entropy_one.html). To show the V3 loop characteristics of these subtypes, the consensus sequence of each subtype was obtained using WebLogo (http://weblogo.threeplusone.com/). Differences in V3 signature pattern between R5 and X4 viruses of each subtype were evaluated using the Los Alamos HIV Sequence Database VESPA (Viral Epidemiology Signature Pattern Analysis) tool (http://www.hiv.lanl.gov/content/sequence/VESPA/vespa.html).

### Env gene cloning, plasmid construction and mutagenesis

The infectious molecular clone pXJDC13 was constructed previously from the HIV-1 CRF07_BC clinical isolate XJDC6291, which was isolated from an IDU in Xijiang Uyghur Autonomous Region of China. XJDC13 is capable of efficient replication in human PBMCs, and displayed CCR5 tropism [32], [35].

The env gene of pXJDC13 was subjected to 35 cycles of PCR amplification. The oligonucleotide primers *Cla*I-1 5′-AGTACAGCTTAATCGATCTGTAGAAATTGTATG-3′ (HxB2 nucleotides [nt] 7079 to 7111) and *Xho*I-2 5′-TCCAGGTCTCGAGATACTGTTCCCACCCCATCTG-3′ (HxB2 nt 8876–8910) were designed to amplify a 1.9-kb envelope fragment containing the C2 region through to the end of gp41. The amplified product was cloned into TA cloning vector pCR-XL-TOPO (Invitrogen) to construct pT-XJDC13V3, and was used as template to perform site-directed mutagenesis by Quickchange II kit (Stratagene). All the V3 mutants were designed according to sequence analysis results and V3 features of CXCR4-tropic viruses reported previously [8], [20], [21], [23]–[25], [36].

Next, mutant V3 loop chimeras were generated by overlapping PCR, and cloned into a pNL4-3 plasmid background to construct infectious molecular clones. The resulting chimeras were confirmed by sequencing to ensure that only the desired substitutions were present.

The 1.9-kb *Cla*I-*Xho*I fragments containing mutagenized V3 loop alleles were excised from pT-XJDC13V3 derived mutants and used to replace the 1.9-kb *Cla*I-*Xho*I fragment of CRF07_BC infectious clone pXJDC13. All mutations in the molecular clone constructs were further verified by DNA sequencing.

For a final set of infectivity assays on a select combination of V3 mutations, additional HIV-1 envelope expression plasmids of subtype C and CRF07_BC were used. Mutations were intruduced into V3 loop of subtype C plasmids C3 (Du156.12), C4 (Du172.17), C12 (ZM55F.PB28a), C13 (ZM109F.PB4), and CRF07_BC plasmids CH70, CH91, CH110, CH119, and CH120.

### Coreceptor usage prediction for HIV-1 CRF07_BC V3 mutants

CRF07_BC V3 mutants were assessed for typical features of X4 viruses, including a V3 net charge of above +5 and basic amino acids at positions 11 and/or 25 (11/25 rule), both of which are predictive of CXCR4 usage [24], [37]. Other features include alteration within the V3 GPGQ crown motif and/or the presence of a two amino acid insertion at positions 13 and 14 [24]. V3 mutants were also predicted by coreceptor genotypic prediction tools, including C-PSSMsinsi (http://indra.mullins.microbiol.washington.edu/webpssm/) [15]; geno2-pheno [coreceptor] (http://coreceptor.bioinf.mpi-inf.mpg.de/) [38]; and CoRSeqV3-C (www.burnet.edu.au/coreceptor) [17].

### Viral preparation and titration

3×10^6^ 293T cells were seeded in a T75 flask and incubated overnight. Monolayers should be 50–80% confluent on the day of transfection. Nine micrograms of wild-type or mutant DNA were transfected into 293T cells by FuGENE 6 according to the manufacturer's instructions (Promega). For pseudoviruses, 4 µg of envelope expression plasmid and 8 µg of backbone plasmid pSG3Δenv were co-transfected into 3×10^6^ 293T cells using FuGENE-6 and incubated at 37°C and 5% CO_2_ for 48 hours. Cell-free supernatants were collected 48 hr after transfection, filtered through 0.45 µm filters, aliquotted and stored at −80°C. For each virus, the p24 concentration was determined by using a commercial ELISA kit and the TCID_50_ titer was evaluated using TZM-bl cells. Briefly, TZM-bl cells were seeded in 96-well plates at a density of 1×10^4^ cells/well in DMEM. Serial dilutions of virus stocks were added in a final volume of 200 µl/well containing 40 µg/ml DEAE-dextran. Fourty-eight hours post-infection, the plates were treated and Relative Light Unit were measured.

### Determination of viral coreceptor utilization

Viral coreceptor utilization was tested on Ghost 3 cells that express CD4 and a specific coreceptor, either CCR5 or CXCR4. These cells contain HIV-2 LTR promoter cassettes that express green fluorescent protein (GFP) in response to stimulation with HIV-1 Tat. Ghost.CD4.CCR5 or Ghost.CD4.CXCR4 cells were seeded in 24-well plates at the density of 6×10^4^ cells/well. On the following day, the monolayers, about 70% confluent, were infected with virus stocks (200 µl/well) in the presence of 20 µg/ml Polybrene to enhance the infective efficiency. After 48 hours, infected cells were detected under fluorescence microscope or harvested and analyzed with flow cytometry (Elite ESP, Beckman Coulter, Germany), and a total of 10,000 to 15,000 events were scored. We expected an approximately 10 fold shift in mean GFP fluorescence of infected cells compared to uninfected cells. The Ghost.CD4.CCR5 or Ghost.CD4.CXCR4 cells infected with Bal or NL4-3 were positive controls, and cells not infected with HIV-1 were the negative control.

### Syncytia assay in MT-2 cells

CXCR4 utilization was confirmed by the appearance of syncytia in MT-2 cells. Infections of MT-2 cells with the CRF07_BC V3 mutants or chimeric viruses were carried out in duplicate in 24-well plates. Briefly, 5×10^4^ MT-2 cells were incubated in 1 ml of the cell-free transfection supernatant containing 1000 TCID_50_ of each virus for 2 h at 37°C and then cultured in 1.5 ml of RPMI 1640 with 10% FBS. Syncytia formation was monitored daily for 7 days.

## Results

### V3 characteristics of HIV-1 subtype B, C and CRF07_BC

**Figure 1:**
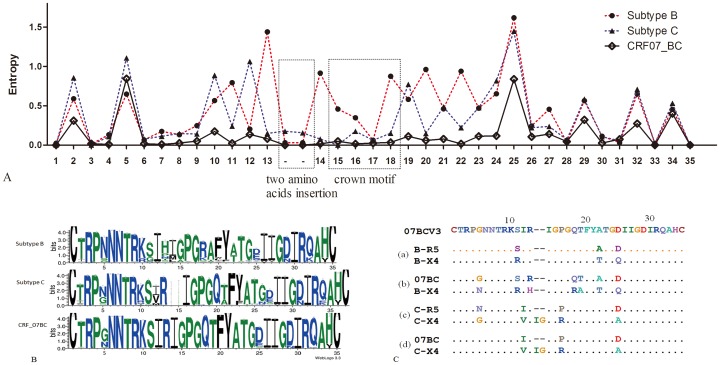
V3 characteristics of HIV-1 subtype B sequences (n = 635, 594 for R5 and 41 for X4), subtype C sequences (n = 359, 339 for R5 and 20 for X4), and CRF07_BC sequences (n = 859). (A) Entropy plot representing the variation at each amino acid site of the V3 loop regions of subtype B, C, and CRF07_BC. The positions of the two amino acid insertion, and the V3 crown motif are indicated. (B) Sequence logos of V3 residues. The character and size of each logo represent the proportion of an amino acid at each site. The sequence logos of subtype B, C, and CRF07_BC were based on 635, 395, and 859 V3 amino acid sequences, respectively. (C) VESPA analysis of V3 sequences. The signature pattern between R5 and X4 viruses in subtype B and C were shown in (a) and (c) respectively. The differences between CRF07_BC viruses and X4 viruses in either subtype B or C are shown in (b) and (d).

The characteristics of the HIV-1 V3 region for three subtypes are shown in Figure 1. Entropy plots depict the amount of variability at each site of an alignment, with higher entropy indicating more variation at a specific site. Subtype B sequences were the most divergent, followed by subtype C, and CRF07_BC sequences were the most conserved (Figure 1A and 1B). Differences between the variability of subtype B and C were mainly between positions 9 and 22. Subtype B V3 sequences were more variable than subtype C in position 11 and the GPGQ crown, and almost no mutations occurred in CRF07_BC sequences at these positions. Sequence variability at position 25 was high in all three subtypes. Two amino acid insertions upstream of the GPGQ crown were present only in subtype C sequences. GPGR was the typical crown tip for subtype B, while GPGQ was typical for subtype C and CRF07_BC.

Sequence analysis results by VESPA showed that in subtype B viruses, the V3 regions of R5 and X4 viruses were significantly different only at three positions, while those in subtype C differed by 5 positions (Figure 1C). In subtype B, compared with R5 V3 sequences (n = 594), the signature pattern of X4 viruses (n = 41) contained Arginine (R), Threonine (T) and Glutamine (Q) substitutions at positions 11, 22 and 25 respectively. In subtype C, compared with the R5 V3 sequences (n = 339), the V3 signature pattern of X4 viruses (n = 20) were Glycine (G), Valine (V), Arginine (R), and Alanine (A) substitutions at positions 5, 12, 16 and 25 respectively, and also contained an insertion of two amino acids (IG) between positions 13 and 14. Most of these coreceptor-specific mutations have been reported previously. In comparison, the X4 V3 sequences for CRF07_BC differed from those of subtype B by seven positions, and from subtype C by five positions (Figure 1C, (b) and (d)).

### Design and coreceptor tropism prediction of CRF07_BC V3 mutants

Based on the sequence analysis above and coreceptor-specific mutations reported previously, we considered the most possible combinations of mutations that determine coreceptor tropism in CRF07_BC, and designed 16 mutants based on the V3 loop of pXJDC13. These mutant amino acid sequences are listed in Table 1. M1 contained three mutations (S11R, A22T and D25Q), which were subtype B X4 signatures (Figure 1C(a)). Arginine substitution at position 11, 18 and/or 25 either alone or in combination was introduced in M2, M3, M4, M5, and M6. Mutants containing the two amino acid IG insertion between positions 13 and 14, either alone or in combination with mutations S11R, I12V, P16R, Q18R and/or D25R, were also designed (M7, M8, M9, M10, M11, M12, M13, M14 and M15). Instead of the IG insertion, M16 contained an MG insertion and arginine substitution at position 11. The net charge of the V3 loops was increased from 4+ to 8+, and their length from 35 aa to 37 aa.

**Table 1:** Coreceptor tropism prediction of CRF07_BC V3 mutants designed in this study.

Mutants	V3	V3 Length	No. of AA changes	Crown	Net charge	Net charge Prediction	11/25	11/25 prediction	C-PSSM prediction	Geno2Pheno prediction	CoRSeqV3-C prediction
pXJDC13	CTRPGNNTRK SIR--IGPGQ TFYATGDIIG DIRQAHC	35	0	GPGQ	4	R5	SD	R5	R5	R5	R5
M1	.......... R..--..... ...T..Q... .......	35	3	GPGQ	6	X4	RQ	X4	X4	X4	X4
M2	.......... R..--..... .......... .......	35	1	GPGQ	5	X4	RD	X4	R5	X4	X4
M3	.......... ...--..... ......R... .......	35	1	GPGQ	6	X4	SR	X4	R5	X4	X4
M4	.......... R..--..... ......R... .......	35	2	GPGQ	7	X4	RR	X4	X4	X4	X4
M5	.......... R..--....R .......... .......	35	2	GPGR	6	X4	RD	X4	X4	X4	X4
M6	.......... R..--....R ......R... .......	35	3	GPGR	8	X4	RR	X4	X4	X4	X4
M7	.......... ...IG..... .......... .......	37	2	GPGQ	4	R5	SD	R5	R5	X4	X4
M8	.......... ...IG....R .......... .......	37	3	GPGR	5	X4	SD	R5	R5	X4	X4
M9	.......... R..IG....R .......... .......	37	4	GPGR	6	X4	RD	X4	X4	X4	X4
M10	.......... ...IG..R.. .......... .......	37	3	GRGQ	5	X4	SD	R5	R5	X4	X4
M11	.......... ...IG..R.. ......R... .......	37	4	GRGQ	7	X4	SR	X4	X4	X4	X4
M12	.......... R..IG..R.. ......R... .......	37	5	GRGQ	8	X4	RR	X4	X4	X4	X4
M13	.......... .V.IG..R.. ......R... .......	37	5	GRGQ	7	X4	SR	X4	X4	X4	X4
M14	.......... RV.IG..R.. .......... .......	37	5	GRGQ	6	X4	RD	X4	X4	X4	X4
M15	.......... RV.IG..R.. ......R... .......	37	6	GRGQ	8	X4	RR	X4	X4	X4	X4
M16	.......... R..MG..... .......... .......	37	3	GPGQ	5	X4	RD	X4	R5	X4	X4

The number of R5 and X4 viruses predicted by each prediction method were as follows: by the net charge rule, 1/16 were R5 and 15/16 were X4 viruses; by the 11/25 rule, 3/16 were R5 and 13/16 were X4 viruses; by C-PSSMsinsi, 6/16 were R5 and 10/16 were X4 viruses; by Geno2pheno_10_, 0/16 were R5 and 16/16 were X4 viruses; by CoRSeqV3-C, 0/16 were R5 and 16/16 were X4 viruses (Table 1). The combined criteria from all these tools predicted that 0/16 were R5 viruses, 6/16 were dual-tropic viruses, and 10/16 were X4 viruses.

### Molecular determinants for coreceptor switching in CRF07_BC V3 loop

To clarify the molecular determinants for coreceptor switching in the CRF07_BC V3 loop, we have characterized a panel of mutant V3 loop chimeras in the genetic background of pNL4-3. After transfection into 293T cells, chimeric viruses containing the V3 mutations were obtained. All 17 mutant V3 chimeric viruses generated in this study were infective in TZM-bl cells and Ghost.CD4+ cells expressing either CCR5 or CXCR4 (Table 2). Compared to chimeric virus with the original V3 loop (NL4-3/pXJDC13V3), the infectivity of viruses containing the two amino acid insertion was decreased by 3- to 258-fold (NL4-3/M7V3 to NL4-3/M16V3). Arginine substitutions at position 11, 18, and 25 also caused NL4-3/M6V3 infectivity to be reduced. However, the infectivity of the remaining five mutant V3 chimeras was increased due to the mutations (NL4-3/M1V3 to NL4-3/M5V3).

**Table 2:** The characteristics of pNL4-3 based chimeras with mutated V3 loops derived from CRF07_BC pXJDC13.

					Syncytia	Ghost.CD4+	
Chimeric viruses	V3	Infectious titer (TCID_50_/ml)	P24 (ng/ml)	Virion infectivity (TCID_50_/ng)	MT-2	CCR5	CXCR4
NL4-3/XJDC13V3	CTRPGNNTRK SIR--IGPGQ TFYATGDIIG DIRQAHC	349386	25.32	13796.10	**−**	**+**	**−**
NL4-3/M1V3	.......... R..--..... ...T..Q... .......	1746928	409.57	4265.27	**−**	**+**	**−**
NL4-3/M2V3	.......... R..--..... .......... .......	1746928	192.10	9093.62	**−**	**+**	**−**
NL4-3/M3V3	.......... ...--..... ......R... .......	1746928	909.48	1920.80	**−**	**+**	**−**
NL4-3/M4V3	.......... R..--..... ......R... .......	1746928	962.18	1815.59	**−**	**+**	**−**
NL4-3/M5V3	.......... R..--....R .......... .......	1746928	399.77	4369.85	**−**	**+**	**−**
NL4-3/M6V3	.......... R..--....R ......R... .......	69877	703.21	99.37	**−**	**+**	**−**
NL4-3/M7V3	.......... ...IG..... .......... .......	13975	355.99	39.26	**−**	**+**	**−**
NL4-3/M8V3	.......... ...IG....R .......... .......	69877	932.99	74.90	**−**	**+**	**−**
NL4-3/M9V3	.......... R..IG....R .......... .......	13975	1030.81	13.56	**−**	**+**	**−**
NL4-3/M10V3	.......... ...IG..R.. .......... .......	2795	522.95	5.34	**+**	**+**	**+**
NL4-3/M11V3	.......... ...IG..R.. ......R... .......	69877	176.93	394.94	**+**	**+**	**+**
NL4-3/M12V3	.......... R..IG..R.. ......R... .......	69877	792.70	88.15	**+**	**+**	**+**
NL4-3/M13V3	.......... .V.IG..R.. ......R... .......	13975	406.96	34.34	**+**	**+**	**+**
NL4-3/M14V3	.......... RV.IG..R.. .......... .......	2795	377.22	7.41	**+**	**+**	**+**
NL4-3/M15V3	.......... RV.IG..R.. ......R... .......	69877	487.01	143.48	**+**	**+**	**+**
NL4-3/M16V3	.......... R..MG..... .......... .......	69877	513.47	136.09	**+**	**+**	**+**

**Figure 2:**
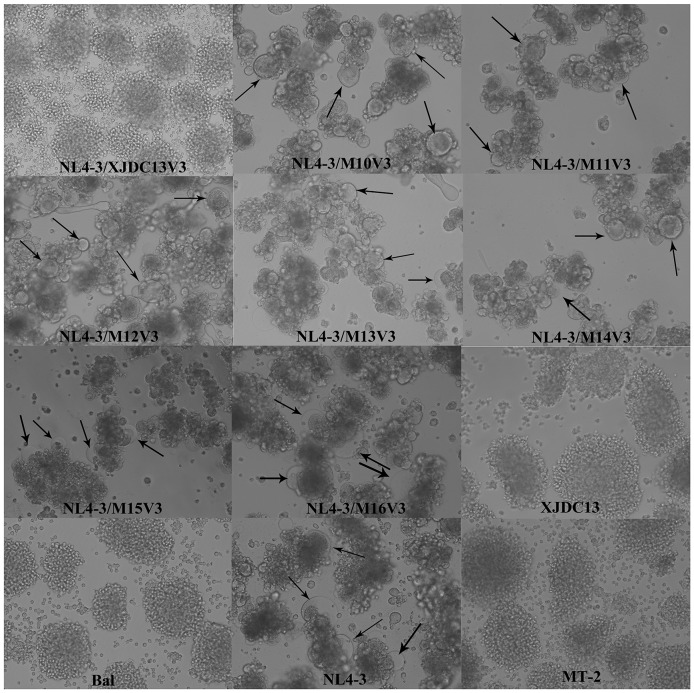
Syncytium formation generated by the infectious molecular clones in MT-2 cells. 5×10^4^ MT-2 cells were incubated in 1 ml of the cell-free transfection supernatant containing 1000 TCID_50_ of each virus in triplicate. Syncytia formation was monitored daily for 7 days. Seven chimeric viruses, which contained V3 loops from M10, M11, M12, M13, M14, M15, and M16, induced syncytium formation in MT-2 cells within five days postinfection, same as NL4-3 (arrows). NL4-3/XJDC13V3, XJDC13, and Bal could not induce syncytium formation in MT-2.

The chimeric virus NL4-3/pXJDC13 carrying the original V3 loop from pXJDC13 was still CCR5-tropic. Subtype B X4-like mutations (S11R, A22T and D25Q) could not confer CXCR4-using characteristics to NL4-3/M1V3. Chimeras containing arginine substitutions at position 11, 18 and/or 25 either alone or in combination could not switch to CXCR4-usage (NL4-3/M2V3 to NL4-3/M6V3). The IG insertion alone or in combination with arginine substitution at position 11 and 18 also could not change chimeras' coreceptor tropism (NL4-3/M7V3 to NL4-3/M9V3). Seven of 17 chimeric viruses were dual-tropic and could generate syncytia in MT-2 cells (Figure 2). Six of the dual-tropic chimeras (NL4-3/M10V3 to NL4-3/M15V3) shared the same mutations (IG insertion and P16R mutation), while another one (NL4-3/M16V3) contained MG insertion and S11R mutation. Therefore, IG insertion and P16R mutation or MG insertion and S11R mutation could confer CRF07_BC V3 with CXCR4-using characteristics.

### V3 mutations were insufficient to confer CXCR4 usage in CRF07_BC genotypes

To create fully functional CRF07_BC X4 variants, the mutated V3 loops were cloned into the pXJDC13 infectious clone to construct V3 mutants in the CRF07_BC genetic background. Arginine substitution at position 11 and/or 25 improved viral infectivity (M2, M3 and M4), but arginine substitutions in combination at position 11, 18, and/or 25 caused viral infectivity to decrease (M5 and M6, Table 3). The M1 mutant carrying subtype B X4-like mutations led to mild reduction in infectivity compared with parental clone. To assess the contributions of V3 mutations in HIV-1 CRF07_BC coreceptor usage, infectivity of the pXJDC13 V3 mutants in Ghost.CD4 cell lines expressing either CCR5 or CXCR4 were assayed by flow cytometry. All the mutants above (M1 to M6) were still CCR5-tropic and could not induce syncytia formation in MT-2 cells.

**Table 3:** The characteristics of HIV-1 CRF07_BC V3 mutants.

					Syncytia	Ghost.CD4+	
Mutants	V3	Infectious titer (TCID_50_/ml)	P24 (ng/ml)	Virion infectivity (TCID_50_/ng)	MT-2	CCR5	CXCR4
pXJDC13	CTRPGNNTRK SIR--IGPGQ TFYATGDIIG DIRQAHC	69877	170.31	410.29	**−**	**+**	**−**
M1	.......... R..--..... ...T..Q... .......	31250	84.44	370.09	**−**	**+**	**−**
M2	.......... R..--..... .......... .......	349386	107.61	3246.69	**−**	**+**	**−**
M3	.......... ...--..... ......R... .......	69877	17.4	4015.87	**−**	**+**	**−**
M4	.......... R..--..... ......R... .......	69877	29.16	2396.1	**−**	**+**	**−**
M5	.......... R..--....R .......... .......	3655	104.95	34.83	**−**	**+**	**−**
M6	.......... R..--....R ......R... .......	559	88.79	6.3	**−**	**+**	**−**
M7	.......... ...IG..... .......... .......	112	197.30	0.57	**−**	**+**	**−**
M8	.......... ...IG....R .......... .......	0	185.06	0	**−**	**−**	**−**
M9	.......... R..IG....R .......... .......	0	102.06	0	**−**	**−**	**−**
M10	.......... ...IG..R.. .......... .......	0	61.37	0	**−**	**−**	**−**
M11	.......... ...IG..R.. ......R... .......	0	164.02	0	**−**	**−**	**−**
M12	.......... R..IG..R.. ......R... .......	0	103.51	0	**−**	**−**	**−**
M13	.......... .V.IG..R.. ......R... .......	0	143.64	0	**−**	**−**	**−**
M14	.......... RV.IG..R.. .......... .......	0	129.30	0	**−**	**−**	**−**
M15	.......... RV.IG..R.. ......R... .......	0	64.59	0	**−**	**−**	**−**
M16	.......... R..MG..... .......... .......	0	331.12	0	**−**	**−**	**−**

The IG insertion caused dramatic reduction in viral infectivity (M7), and more mutations in combination with this insertion abrogated viral infectivity completely (M8, M9, M10, M11, M12, M13, M14 and M15). Also, the M16 mutant, which contained an MG insertion and an S11R mutation, was non-infectious. V3 loops from M10, M11, M12, M13, M14, M15 and M16 conferred pNL4-3-based chimeras CXCR4-using characteristics, but these mutations abrogated viral infectivity in the pXJDC13 genetic background.

### V3 mutations have similar effects in other env genotypes in HIV-1 subtype C and CRF07_BC

Finally, we assessed whether IG or MG insertion, combined with S11R mutation (RMG), will exert the same influence on viral infectivity in the context of other subtype C and CRF07_BC envelope gene backbones. We constructed V3 mutant pseudoviruses with these mutations using HIV-1 envelope expression plasmids, four for subtype C and five for CRF07_BC (Table 4). The infectivity of subtype C pseudoviruses with the IG insertion or RMG mutation decreased dramatically. All the CRF07_BC pseudoviruses with RMG mutation were defective in infectivity, and the pseudoviruses with IG insertion were also non-infectious or poor in infectivity. These mutations did not confer CXCR4-using ability, and the infection-competent pseudoviruses were still CCR5-tropic. Notably, additional mutations were also introduced unintentionally along with these insertions, such as V12I for C3 and C4 clones, L14I for C13 clone and G13R for CH70 clones.

**Table 4:** The impact of RMG or IG insertion on HIV-1 subtype C and CRF07_BC envelopes.

						Ghost.CD4+	
Subtype	Mutants	V3	Infectious titer (TCID_50_/ml)	P24 (ng/ml)	Virion infectivity (TCID_50_/ng)	CCR5	CXCR4
	pXJDC13	**CTRPGNNTRKSIR--IGPGQTFYATGDIIGDIRQAHC**	69877	170.31	410.29	**+**	**−**
C	C3	**....N......V.--......................**	13975	78.34	178.39	**+**	**−**
	C3-RMG	**....N.....R..MG......................**	112	120.96	0.93	**+**	**−**
	C4	**....S......V.--.......F..............**	13975	82.95	168.48	**+**	**−**
	C4-RMG	**....S.....R..MG......................**	112	63.3	1.77	**+**	**−**
	C4-IG	**....S........IG......................**	112	137.37	0.82	**+**	**−**
	C12	**.............--.....A.F..TN........Y.**	349386	145.38	2403.31	**+**	**−**
	C12-RMG	**..........R..MG..........TN........Y.**	112	282.71	0.4	**−**	**−**
	C13	**.I...........--L...........V.....K.Y.**	13975	41.06	340.34	**+**	**−**
	C13-RMG	**.I........R..MG............V.....K.Y.**	112	73.41	1.53	**+**	**−**
	C13-IG	**.I...........IG............V.....K.Y.**	2795	43.89	63.68	**+**	**−**
CRF07_BC	CH070	**..........G.G--...........E......K...**	349386	57.64	6061.59	**+**	**−**
	CH70-RMG	**..........R..MG...........E......K...**	0	111.58	0	**−**	**−**
	CH70-IG	**.............IG...........E......K...**	0	124.59	0	**−**	**−**
	CH119	**....N........--......................**	349386	78.99	4423.31	**+**	**−**
	CH119-RMG	**....N.....R..MG......................**	0	151.52	0	**−**	**−**
	CH119-IG	**....N........IG......................**	112	75.19	1.49	**+**	**−**
	CH91	**....N........--......................**	69877	69.61	1003.88	**+**	**−**
	CH91-IG	**....-........IG......................**	0	148.61	0	**−**	**−**
	CH110	**.I..N........--......................**	69877	78.66	888.30	**+**	**−**
	CH110-IG	**.I..N........IG......................**	112	112.55	1.00	**+**	**−**
	CH110-RMG	**.I..N.....R..MG......................**	112	201.68	0.56	**−**	**−**
	CH120	**....N........--...........E..........**	349386	81.25	4300.05	**+**	**−**
	CH120-IG	**....N........IG...........A..........**	112	163.62	0.68	**−**	**−**

## Discussion

To our knowledge, this study is the largest and most comprehensive analysis to date on the characteristics of the V3 loop of HIV-1 CRF07_BC. To clarify the molecular determinants of coreceptor switching in this genotype, we designed a panel of mutants based on the V3 loop of CRF07_BC infectious clone pXJDC13. We constructed and characterized 17 chimeric viruses with these mutated V3 loops in the genetic background of pNL4-3. Chimeric virus containing the original V3 loop from pXJDC13 was still CCR5-tropic, and seven mutant V3 chimeric viruses were dual-tropic and could induce syncytia formation in MT-2 cells (Table 2). Six of seven dual-tropic mutants (NL4-3/M10V3 to NL4-3/M15V3) shared the same mutations (IG insertion and P16R mutation), and another one (NL4-3/M16V3) contained MG insertion and S11R mutation. Therefore, IG insertion and P16R or MG insertion and S11R mutation could confer CRF07_BC V3 with CXCR4-using characteristics. However, IG insertion either alone or in combination with Q18R and/or S11R was unable to change viral coreceptor tropism (NL4-3/M7V3 to NL4-3/M9V3).

The subtype B X4 V3 features could not confer mutants (NL4-3/M1V3 to NL4-3/M6V3) CXCR4-using ability and the mutants (NL4-3/M10V3 to NL4-3/M16V3) designed according to subtype C X4 features reported recently [25], [36], could use CXCR4 as coreceptor. CRF07_BC and subtype C viruses share the same characteristics in CXCR4-usage, which may be due to the fact that CRF07_BC envelope region is derived from subtype C.

In order to construct CRF07_BC X4 variants, we created 16 V3 mutants based on the pXJDC13 backbone. In previous studies for subtype B, mutations such as S11R, A22T, and D25Q or D25R alone within the V3 were the minimal requirement for production of SI viruses [20], [21]. However, in our study, these mutations could not confer CRF07_BC with CXCR4-using ability (M1). Similarly, basic amino acid substitutions at position 11 and/or 25 are known to be strong determinants of CXCR4-mediated entry [22], but we found that arginine substitutions at positon 11, 18 and/or 25 either alone or in combination also could not alter viral coreceptor tropism (M2, M3, M4, M5 and M6, Table 3). The mutant V3 chimeric viruses containing these mutations were also CCR5 tropic (Table 2). Insertion of two amino acids prior to the crown is also known as a highly sensitive characteristic for predicting subtype C CXCR4-using viruses [26], [36]. Residue changes in three positions of subtype C V3 domain are critical for the dual tropic phenotype, which include substitution of arginine at position 11, MG or LG insertion between positions 13 and 14, and substitution of threonine at the position immediately downstream of the GPGQ crown [25]. However, we found that these changes in the CRF07_BC V3 loop (M16) abolished viral infectivity. The IG insertion (M7) caused viral infectivity to decrease dramatically and did not confer CXCR4-using characteristics. More mutations based on M7 at position 11, 12, 16, 18, and/or 25 (M8, M9, M10, M11, M12, M13, M14, and M15) completely abrogated viral infectivity. Finally, the IG insertion and P16R mutation, either alone or in combination, is known to render subtype C Env completely non-functional for HIV-1 entry into cells expressing either CCR5 or CXCR4, and additional mutations in V1 and/or V3 was likely be required to exert an influence on CXCR4 usage [36]. Here, the V3 loops from mutants M10, M11, M12, M13, M14 and M15, and M16 were functional in the pNL4-3 backbone and confered chimeric viruses with CXCR4-using ability, but were non-functional in the CRF07_BC pXJDC13 genetic background. These results suggest that the effects of IG insertion and P16R mutation or MG insertion and S11R mutation on CXCR4 usage are context dependent, and additional mutations are needed to compensate for these fitness-reducing alterations.

To determine whether IG insertion or RMG mutation will exert the same influence on other envelopes of subtype C and CRF07_BC, we have created two panels of V3 mutants based on envelope expression clones. The RMG mutation caused dramatic decrease in the infectivity of four subtype C clones and one CRF07_BC clone, and completely abrogated the infectivity of two CRF07_BC clones (Table 4). Similarly, the IG insertion diminished the infectivity in two subtype C clones and two CRF07_BC clones, and abolished the infectivity of two CRF07_BC clones. All the infectious pseudoviruses were still CCR5-tropic. It seems that these insertions alone could not confer the mutants with CXCR4-using ability, and instead decreased or abrogated infectivity in subtype C and CRF07_BC envelopes.

As in previous studies, it is reasonable to characterize the V3 features of coreceptor switching variants in subtype B CXCR4-using background, such as NL4-3 and HxB2 [20], [21], [39], and this eliminated the impact of other regions outside V3 on viral infectivity. Besides V3, mutations in V1/V2, C2, V4, V5, or C4 may be necessary to maintain viral fitness [8], [36], [40]–[44], and gp41 sequences may also influence viral entry mediated by CCR5 or CXCR4 [45], [46]. In supplement to our findings above, we have also constructed 4 chimeric viruses containing the V3, V1–V3, V3–V5, or V1–V5 regions of pNL4-3 within the pXJDC13 backbone, and all these chimeric viruses were defective in infectivity (data not shown). The sequence diversity between subtype B and CRF07_BC envelope regions may be too great, and backbone incompatibility may be an underlying cause for the defectiveness of these chimeras. Further studies are needed to clarify the mechanisms of fitness compensation involved in coreceptor switching. One approach can be to try and construct true CRF07_BC CXCR4-using viruses by exchanging different regions of the envelope gene with that of subtype C X4 viruses, in order to determine the compensatory sites for coreceptor switching as well as the R5-to-X4 evolutional pathway in CRF07_BC.

In conclusion, we have characterized V3 loop features in HIV-1 CRF07_BC strains capable of conferring CXCR4 usage in pNL4-3 background, which shared the same features with V3 in subtype C X4 viruses. However, all CXCR4-tropic V3 loops completely abolished mutant viruses infectivity in the CRF07_BC strain genetic background offered by molecular clone pXJDC13. These results suggest that alterations in the V3 loop are necessary to confer CXCR4 usage, but compensatory mutations elsewhere in the envelope are needed to maintain viral fitness. Understanding the coreceptor tropism of HIV-1 CRF07_BC will be critical for improving targeted treatment and vaccine development in China.
